# Impact of Rotavirus Vaccine Introduction in Children Less Than 2 Years of Age Presenting for Medical Care With Diarrhea in Rural Matlab, Bangladesh

**DOI:** 10.1093/cid/ciz133

**Published:** 2019-02-12

**Authors:** Lauren M Schwartz, K Zaman, Md Yunus, Ahasan-ul H Basunia, Abu Syed Golam Faruque, Tahmeed Ahmed, Mustafizur Rahman, Jonathan D Sugimoto, M Elizabeth Halloran, Ali Rowhani-Rahbar, Kathleen M Neuzil, John C Victor

**Affiliations:** 1 Department of Epidemiology, School of Public Health, University of Washington, Seattle; 2 Vaccine and Infectious Diseases Division, Fred Hutchinson Cancer Research Center, Seattle; 3 International Centre for Diarrhoeal Disease Research, Bangladesh, Dhaka; 4 Department of Biostatistics, School of Public Health, University of Washington, Seattle; 5 Center for Inference and Dynamics of Infectious Diseases, Seattle; 6 Center for Vaccine Development, University of Maryland School of Medicine, Baltimore; 7 Center for Vaccine Innovation and Access, PATH, Seattle, Washington

**Keywords:** rotavirus vaccine, impact, time-series

## Abstract

**Background:**

Following the conclusion of a human rotavirus vaccine (HRV) cluster-randomized, controlled trial (CRT) in Matlab, Bangladesh, HRV was included in Matlab’s routine immunization program. We describe the population-level impact of programmatic rotavirus vaccination in Bangladesh in children <2 years of age.

**Methods:**

Interrupted time series were used to estimate the impact of HRV introduction. We used diarrheal surveillance collected between 2000 and 2014 within the 2 service delivery areas (International Centre for Diarrhoeal Disease Research, Bangladesh [icddr,b] service area [ISA] and government service area [GSA]) of the Matlab Health and Demographic Surveillance System, administered by icddr,b. Age group–specific incidence rates were calculated for both rotavirus-positive (RV+) and rotavirus-negative (RV–) diarrhea diagnoses of any severity presenting to the hospital. We used 2 models to assess the impact within each service area: Model 1 used the pre-vaccine time period in all villages (HRV– and control-only) and Model 2 combined the pre-vaccine time period and the CRT time period, using outcomes from control-only villages.

**Results:**

Both models demonstrated a downward trend in RV+ diarrheal incidences in the ISA villages during 3.5 years of routine HRV use, though only Model 2 was statistically significant. Significant impacts of HRV on RV+ diarrhea incidences in GSA villages were not observed in either model. Differences in population-level impacts between the 2 delivery areas may be due to the varied rotavirus vaccine coverage and presentation rates to the hospital.

**Conclusions:**

This study provides initial evidence of the population-level impact of rotavirus vaccines in children <2 years of age in Matlab, Bangladesh. Further studies are needed of the rotavirus vaccine impact after the nationwide introduction in Bangladesh.

## Teaser

A time-series analysis suggests a downward trend in rotavirus diarrhea in children following the rotavirus vaccine’s introduction in Matlab, Bangladesh. Further research is needed to confirm the findings and to increase support for the introduction of rotavirus vaccines in Asia.

**(See the Editorial Commentary by Steele and Parashar on pages 2071–3.)**

Globally, an estimated 13 000 deaths due to rotavirus diarrhea occur annually in children <5 years of age, with most of the burden in sub-Saharan Africa and Asia [1]. While diarrhea-associated mortality rates have decreased globally in the last decade, the burden of rotavirus diarrhea remains substantial in low-income settings [2]. In 2006, 2 rotavirus vaccines were introduced worldwide: GlaxoSmithKline’s human rotavirus vaccine (HRV; Rotarix) and Merck’s pentavalent rotavirus vaccine (PRV; RotaTeq). Large, multi-site, randomized, controlled trials (RCTs) of both vaccines in Africa demonstrated moderate vaccine efficacy (VE) against severe rotavirus diarrhea during the first year of life [3, 4]. As of August 2018, 96 countries, of which 46 are Gavi-eligible, have introduced rotavirus vaccines into their regional or national immunization programs [5]. In the World Health Organization (WHO) Africa region, 74% of countries have introduced rotavirus vaccination. Studies in sub-Saharan Africa have shown statistically significant rotavirus vaccine effectiveness and population-level impacts against all-cause and rotavirus diarrhea in children <5 years of age within 2–3 years of the initiation of routine use [6–14].

Despite the WHO recommendation for rotavirus vaccine use worldwide, only 18% of countries in the WHO southeast Asia region have introduced a rotavirus vaccine [5]. Limited data on vaccine effectiveness and population impacts may have slowed the introduction of rotavirus vaccines in Asia [15]. The only multi-site RCT of PRV in Asia demonstrated moderate vaccine efficacy against severe rotavirus gastroenteritis in the first 2 years of life (Bangladesh VE 42.7%, 95% confidence interval [CI] 10.4–63.9; Vietnam VE 63.9%, 95% CI 7.6–90.9; combined VE 51.0%, 95% CI 12.8–73.3) [16]. In Bangladesh, this RCT included half of the Matlab villages (International Centre for Diarrhoeal Disease Research, Bangladesh [icddr,b] service areas).

To evaluate the effectiveness of HRV on rotavirus diarrhea in Asia, a 2-year cluster-randomized trial (CRT) was conducted in all villages in Matlab, Bangladesh, beginning in 2008 [17]. The overall effectiveness, which assesses the overall reduction in the incidence of acute rotavirus diarrhea, regardless of vaccination status, was 29.0% (95% CI 11.3–43.1) in children <2 years of age. This study provided initial evidence of the potential population impact of routine rotavirus vaccine use in Bangladesh. After the CRT, HRV was provided for routine use among infants in all Matlab villages between March 2011 and September 2014.

To evaluate the population-level impact of HRV in Matlab, Bangladesh, during the 3.5 years of routine use following the CRT, we examined trends in the rotavirus-positive (RV+) and rotavirus-negative (RV–) diarrhea incidence rates of any severity presenting to Matlab Hospital between February 2000 and September 2014.

## METHODS

### Study Setting

The study utilized diarrheal surveillance data collected among children <2 years of age residing in villages of the Matlab Health and Demographic Surveillance System (HDSS), administered by the icddr,b, and presenting to Matlab Hospital [18]. The HDSS is divided into the icddr,b service area (ISA; 67 villages) and the government service area (GSA; 75 villages). The icddr,b provides ISA villages with child and maternal health intervention programs and the Bangladesh Ministry of Health and Family Welfare provides GSA villages with the government standard of care. The HDSS maintains a census and registration of vital events, including internal and external migration.

### Immunization Records

The HDSS also maintains immunization records through a formal record-keeping system. In the ISA villages, community health workers maintain vaccination records, and in the GSA villages, community health workers check vaccination cards or ask mothers if the card is missing.

### Diarrheal Surveillance

Matlab Hospital is the central diarrhea treatment facility for the Matlab HDSS population. This study includes data from children <2 years of age. The incidence rate for presentations to Matlab Hospital of all-cause diarrhea among children from GSA villages has historically been about half of the incidence rate for presentations from ISA villages [17]. Stool specimens are collected from all patients presenting with diarrhea (3 or more loose stools per 24 hours) to Matlab Hospital. The samples are tested for group A rotavirus VP6 antigens using a solid-phase, sandwich-type enzyme immunoassay (Prospect, Oxoid Diagnostics Ltd, Hampshire, United Kingdom).

### Statistical Analysis

Interrupted time series, using segmented regression models, were used to estimate the impact of the rotavirus vaccine introduction in Matlab, Bangladesh, among children <2 years of age [19]. The monthly incidence rates of RV+ and RV– diarrhea were examined separately, by age group (0 to <12 months, 12 to <24 months, and combined [0 to <24 months]). The incidence rates were calculated for RV+ and RV– diarrhea with the number of events presenting to Matlab Hospital per month as the numerator and the monthly population at risk, using HDSS census estimates, as the denominator.

Due to varied rotavirus vaccine coverage and baseline diarrheal incidences, analyses were conducted separately for the ISA and GSA villages.

Among the ISA villages, the pre-vaccine time period was defined as February 2000–February 2007; the RCT period as March 2007–March 2009); the CRT period as April 2009–March 2011; and the HRV introduction period as April 2011–September 2014. Among the GSA villages, the pre-vaccine time period was defined as February 2000–October 2008; the CRT period as November 2008–March 2011; and the HRV introduction period as April 2011–September 2014. During the CRT periods, the villages were stratified by service area and then randomized to control-only (no placebo) or HRV.

We used 2 models to estimate the impact of HRV use on RV+ and RV– diarrhea incidence rates. Model 1 was defined *a priori*, while Model 2 was defined after examining the count data. Model 1 and Model 2 differ by both the baseline period used as the referent category and the types of villages included (HRV– and/or control-only). In both models, a generalized linear model was fit to the time-series data, assuming a negative, binomial distribution due to over-dispersion of the data [20]. Calendar months were included in each model to account for seasonality, and a sequential, monthly term for every month over the entire time period was included to account for secular trends. The natural log of the monthly population at risk was included in the model as the offset term. The Breusch-Godfrey test identified some autocorrelation; therefore, 95% CIs were estimated using Newey-West heteroskedastic- and autocorrelation-consistent variance estimators, with a lag of 2 [19, 21]. The estimates of the coefficients for each time period were exponentiated to estimate incidence rate ratios (IRRs), compared to the referent category.

In Model 1, within the ISA and GSA areas separately, the corresponding pre-vaccine time period was used as the referent category. Villages randomized as both HRV and control-only were included in the analysis. To estimate the IRRs and corresponding 95% CIs, the time periods corresponding to the RCT, CRT, and each of the 3.5 years of routine HRV use were modeled with separate indicator variables. This is a conservative model, which directly compares incidence rates in February 2000–February 2007 (ISA villages) and February 2000–October 2008 (GSA villages) to the years of routine HRV use, starting in April 2011 in all ISA and GSA villages.

In the secondary analysis (Model 2), only the villages randomized as control-only during the CRT were used. Within the ISA and GSA regions, the pre-vaccine and CRT time periods were combined in the referent category. The time period corresponding to the RCT was excluded. To estimate the IRRs and corresponding 95% CIs, each of the 3.5 years of routine HRV use were modeled with separate indicator variables. This approach directly compared incidence rates in February 2000–March 2011, excluding the RCT time period, to the years of routine HRV use, starting in April 2011 in those ISA and GSA villages randomized as controls.

The monthly vaccine coverage was estimated as the proportion of children 6 to <52 weeks old receiving each HRV dose within regions of Matlab, Bangladesh. Analyses were completed using Stata version 14 (Stata Corporation, College Station, TX). This study was approved by the ethical review committee of icddr,b in Bangladesh and the Fred Hutchinson Cancer Research Center.

## RESULTS

**Figure 1.**
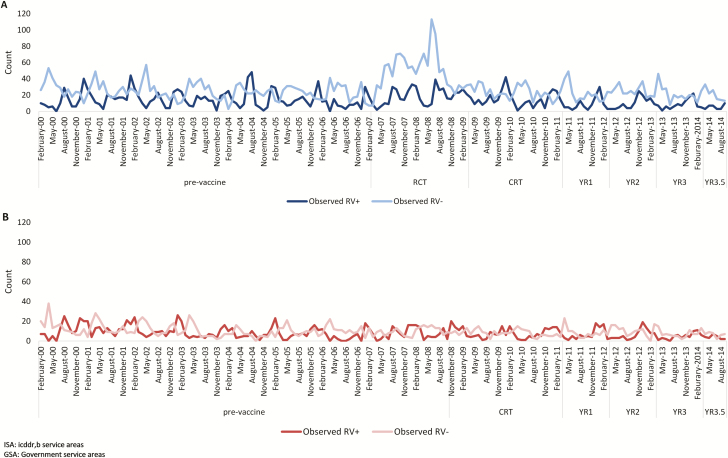
Observed counts of rotavirus-positive (RV+) and rotavirus-negative (RV–) diarrhea of any severity, presenting to Matlab Hospital in (*A*) ISA and (*B*) GSA areas. Abbreviations: CRT, cluster-randomized controlled trial; GSA, government service area; ISA, International Centre for Diarrhoeal Disease Research, Bangladesh, service area; RCT, randomized, controlled trial; YR, year.

Tables 1 and 2 and Figure 1 show RV+ and RV– counts and average incidence rates over time in the GSA and ISA villages, using the study populations used for Models 1 and 2.

**Table 1.** Trends in Diarrhea Presenting to Matlab Hospital by Time Period and Model in International Centre for Diarrhoeal Disease Research, Bangladesh, Service Area

	Model 1								Model 2					
ISA	February 2000– February 2007 (prevaccine)	March 2007– March 2009 (RCT)	April 2009– March 2011 (CRT)	April 2011– March 2012 (YR1)	April 2012– March 2013 (YR2)	April 2013– March 2014 (YR3)	April 2014– September 2014 (YR3.5)	Vaccine Years (April 2011– September 2014)	February 2000– March 2011 (prevaccine and CRT, exclude RCT)	April 2011– March 2012 (YR1)	April 2012– March 2013 (YR2)	April 2013– March 2014 (YR3)	April 2014– September 2014 (YR3.5)	Vaccine Years (April 2011– September 2014)
*0–12 months of age*														
Population	18 281	5153	4936	2494	2683	2586	1286	9048	12 119	1327	1427	1342	681	4777
RV+, count	738	265	216	64	81	54	24	223	511	41	47	33	15	136
RV–, count	1258	823	380	179	208	166	79	632	865	92	122	97	44	355
RV+ incidence	40	51	44	26	30	21	19	25	42	31	33	25	22	28
RV– incidence	69	160	77	72	78	64	61	70	71	69	85	72	65	74
*12–24 months of age*														
Population	18 363	5124	5008	2437	2493	2649	1275	8853	12 243	1280	1325	1408	661	4674
RV+, count	502	200	145	43	41	49	9	142	351	27	20	28	4	79
RV–, count	844	432	185	87	90	89	44	310	558	46	47	53	24	170
RV+ incidence	27	39	29	18	16	19	7	16	29	21	15	20	6	17
RV– incidence	46	84	37	36	36	34	35	35	46	36	35	38	36	36
*0–24 months of age*														
Population	36 644	10 276	9945	4930	5176	5235	2561	17 901	24 363	2607	2752	2750	1342	9451
RV+, count	1240	465	361	107	122	103	33	365	862	68	67	61	19	215
RV–, count	2102	1255	565	266	298	255	123	942	1423	138	169	150	68	525
RV+ incidence	34	45	36	22	24	20	13	20	35	26	24	22	14	23
RV– incidence	57	122	57	54	58	49	48	53	58	53	61	55	51	56

Incidence data are per 1000 person-years.

Abbreviations: CRT, cluster-randomized controlled trial; ISA, International Centre for Diarrhoeal Disease Research, Bangladesh, service area; RCT, randomized, controlled trial; RV–, rotavirus negative; RV+, rotavirus positive; YR, year.

**Table 2.** Trends in Diarrhea Presenting to Matlab Hospital by Time Period and Model in Government Service Area

	Model 1							Model 2					
GSA	February 2000- October 2008 (prevaccine)	November 2008-March 2011 (CRT)	April 2011– March 2012 (YR1)	April 2012– March 2013 (YR2)	April 2013– March 2014 (YR3)	April 2014– September 2014 (YR3.5)	Vaccine Years (April 2011–September 2014)	February 2000– March 2011 (prevaccine and CRT)	April 2011– March 2012 (YR1)	April 2012– March 2013 (YR2)	April 2013– March 2014 (YR3)	April 2014– September 2014 (YR3.5)	Vaccine Years (April 2011–September 2014)
*0–12 months of age*													
Population	22 777	5359	2317	2306	2201	1162	7987	12 163	962	988	934	483	3368
RV+, count	542	144	35	51	37	15	138	285	21	18	14	5	58
RV–, count	671	142	59	80	64	26	229	310	27	32	24	11	94
RV+ incidence	24	27	15	22	17	13	17	23	22	18	15	10	17
RV– incidence	29	26	25	35	29	22	29	25	28	32	26	23	28
*12–24 months of age*													
Population	23 168	5641	2224	2312	2305	1111	7951	12 507	939	970	993	479	3381
RV+, count	365	90	46	28	20	8	102	196	25	12	12	1	50
RV–, count	459	94	40	44	24	13	121	249	15	11	11	5	42
RV+ incidence	16	16	21	12	9	7	13	16	27	12	12	2	15
RV– incidence	20	17	18	19	10	12	15	20	16	11	11	10	12
*0–24 months of age*													
Population	45 945	10 999	4541	4618	4506	2273	15 938	24 670	1901	1958	1927	963	6748
RV+, count	907	234	81	79	57	23	240	481	46	30	26	6	108
RV–, count	1130	236	99	124	88	39	350	559	42	43	35	16	136
RV+ incidence	20	21	18	17	13	10	15	19	24	15	13	6	16
RV– incidence	25	21	22	27	20	17	22	23	22	22	18	17	20

Incidence data are per 1000 person-years.

Abbreviations: CRT, cluster-randomized controlled trial; GSA, government service area; RV–, rotavirus negative; RV+, rotavirus positive; YR, year.

### Rotavirus Vaccine Coverage and Timing

Rotavirus vaccine was not available in Matlab between February 2000 and March 2007. Between April 2007 and March 2009, 568 infants in ISA villages were randomized to receive PRV and 568 infants were randomized to placebo as part of a multi-site RCT [16]. In the stratified HRV CRT in both ISA and GSA areas, villages were randomized to 2 doses of HRV at 6 and 10 weeks of age or randomized as observed, control-only villages [17]. In the GSA villages, the CRT started in November 2008, and in the ISA villages, the CRT started in April 2009. Follow-ups and vaccinations during the CRT occurred in both the ISA and GSA villages through March 2011. Through a donation of vaccines post-CRT, HRV was provided routinely starting in April 2011. After September 2014, the rotavirus vaccine was unavailable.

**Figure 2.**
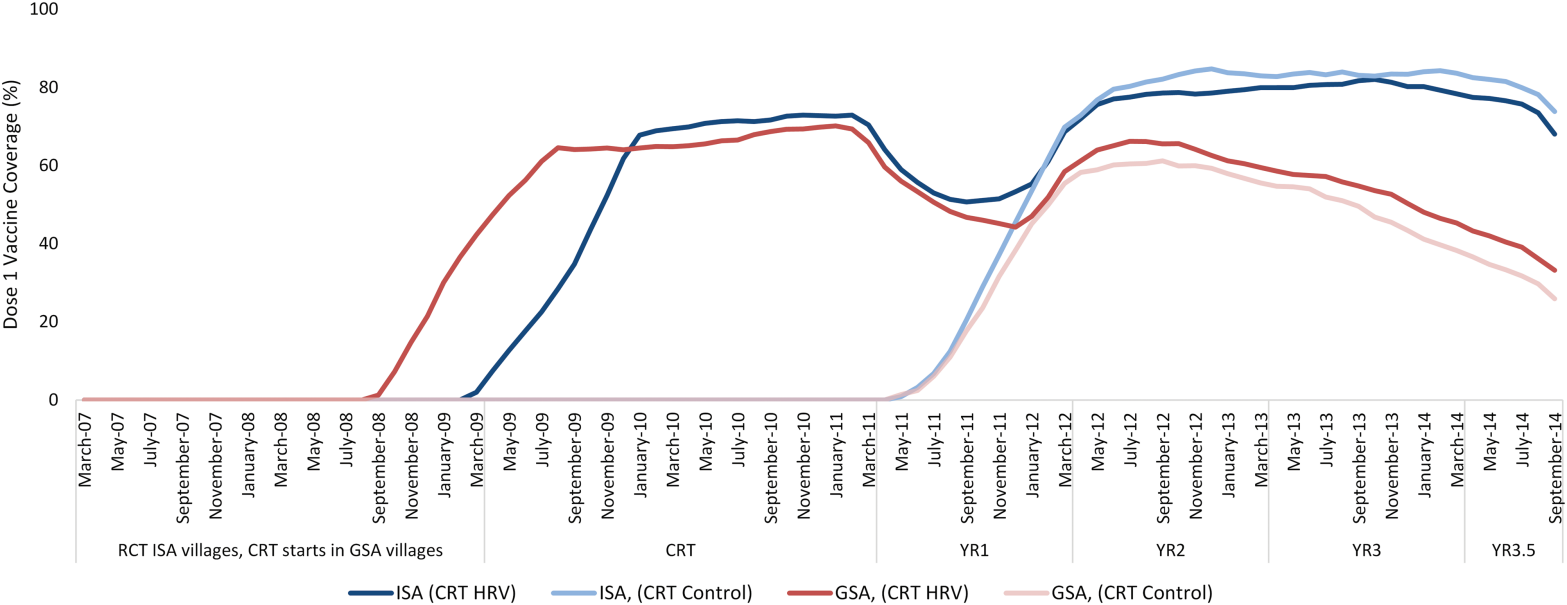
Timing of HRV coverage (dose 1) over time by ISA and GSA villages randomized to HRV or control only in <1-year-olds. Abbreviations: GSA, government service area; HRV, human rotavirus vaccine; ISA, International Centre for Diarrhoeal Disease Research, Bangladesh, service area. ISA, HRV: icddr, b service areas randomized to HRV during the CRT; ISA, Control: icddr, b service areas randomized as control-only villages during the CRT; GSA, HRV: Government service areas randomized to HRV during the CRT; GSA, Control: icddr, b service areas randomized as control-only villages during the CRT; *23 children were vaccinated in GSA Villages in September-October 2008 before the start of the cluster-randomized trial (CRT). This time period is still considered prevaccine due to the small number of children vaccinated.

HRV vaccine coverage levels among children <1 year of age changed during the study period (Figure 2). During the CRT, both the ISA and GSA villages showed similar vaccine coverage levels. Following the CRT, the coverage level among age-eligible children in ISA villages was maintained at between 65–80%, while GSA villages decreased to 42% at the end of the study period.

Observed and predicted RV+ diarrhea counts in ISA and GSA villages for both models demonstrated a satisfactory model fit (Supplementary Figures 1–2).

### Diarrhea Incidence Trends: International Centre for Diarrhoeal Disease Research, Bangladesh, Service Area Villages

**Figure 3.**
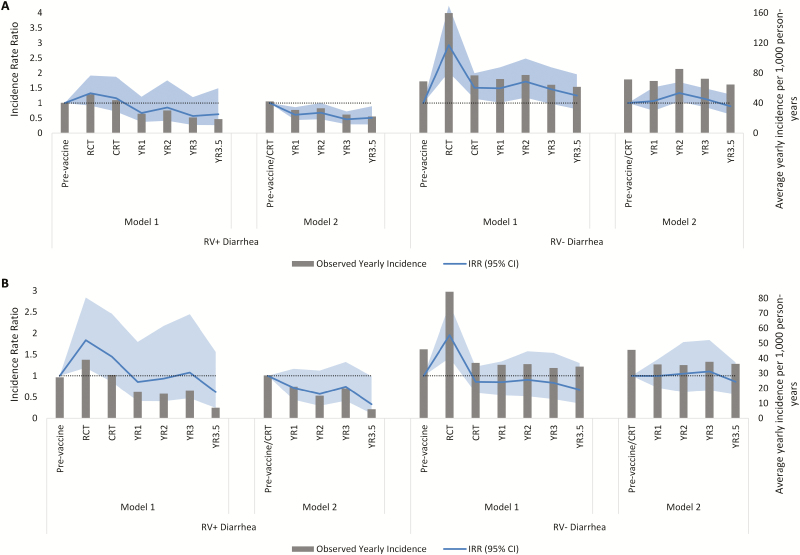
**Observed incidences and** IRRs **of RV+ and RV– diarrhea of any severity presenting to Matlab Hospital in ISA villages using Models 1 and 2 in (*A*) 0– to <**12-month-old children **and (*B*) 12 to <**24-month-old children. Abbreviations: CI, confidence interval; CRT, cluster-randomized controlled trial; IRR, incidence rate ratio; ISA, International Centre for Diarrhoeal Disease Research, Bangladesh, service area; RV–, rotavirus negative; RV+, rotavirus positive; RCT, randomized, controlled trial; YR, year.

Using Model 1, with the pre-vaccine time period as the referent category, RV+ diarrhea rates increased during the RCT period and the CRT period in both age groups in ISA villages (Table 3; Figure 3). During periods of routine HRV use, there was a downward trend that was not statistically significant in RV+ diarrhea incidences after each additional year of vaccine use. During the entire 3.5 years of routine use, there was no meaningful decrease in RV+ diarrhea rates in 0- to <12-month-old children (IRR 0.72, 95% CI 0.39–1.33) or 12- to <24-month-old children (IRR 0.91, 95% CI 0.46–1.83). Using Model 2, combining the pre-vaccine time period and the CRT time period in the reference category and using control-only villages, there was a downward trend in the RV+ diarrhea incidence rates after each additional year of routine HRV use in both age groups (Table 4; Figure 3). During 3.5 years of routine HRV use, there was a statistically significant, 41% decrease in RV+ diarrhea rates in 0- to <12-month-old children (IRR 0.59, 95% CI 0.43–0.80), a 35% decrease in 12- to <24-month-old children (IRR 0.65, 95% CI 0.42–1.02), and a statistically significant, 39% decrease in children 0 to <24 months of age (IRR 0.61, 95% CI 0.45–0.82).

**Table 3.** Diarrhea Trends in International Centre for Diarrhoeal Disease Research, Bangladesh, Service Area Region (Model 1)

ISA	Feb 2000- Feb 2007 (prevaccine)	March 2007– March 2009 (RCT)			April 2009– March 2011 (CRT)			April 2011– March 2012 (YR1)			April 2012– March 2013 (YR2)			April 2013– March 2014 (YR3)			April 2014– September 2014 (YR3.5)			April 2011– September 2014 (vaccine years)		
		IRR	95%	CI	IRR	95%	CI	IRR	95%	CI	IRR	95%	CI	IRR	95%	CI	IRR	95%	CI	IRR	95%	CI
*0–12 months of age*																						
RV+	REF	1.32	0.91	1.92	1.16	0.72	1.88	0.67	0.37	1.21	0.85	0.41	1.75	0.57	0.27	1.20	0.63	0.26	1.49	0.72	0.39	1.33
RV–	REF	2.93	2.03	4.24	1.51	1.14	2.00	1.49	1.01	2.19	1.71	1.18	2.49	1.46	0.97	2.19	1.25	0.79	1.96	1.59	1.09	2.31
*12–24 months of age*																						
RV+	REF	1.84	1.19	2.84	1.45	0.86	2.46	0.86	0.41	1.80	0.94	0.40	2.17	1.08	0.47	2.45	0.62	0.25	1.56	0.91	0.46	1.83
RV–	REF	1.95	1.42	2.69	0.86	0.60	1.22	0.85	0.54	1.34	0.91	0.52	1.58	0.83	0.45	1.54	0.67	0.35	1.30	0.88	0.56	1.38
*0–24 months of age*																						
RV+	REF	1.50	1.03	2.19	1.26	0.80	2.00	0.72	0.40	1.31	0.90	0.44	1.84	0.74	0.36	1.52	0.66	0.29	1.51	0.79	0.43	1.43
RV–	REF	2.55	1.91	3.41	1.24	0.98	1.55	1.23	0.89	1.70	1.40	0.99	1.97	1.19	0.82	1.73	1.01	0.68	1.49	1.31	0.95	1.79

Abbreviations: CI, confidence interval; CRT, cluster-randomized controlled trial; IRR, incidence rate ratio; ISA, International Centre for Diarrhoeal Disease Research, Bangladesh, service area; RCT, randomized, controlled trial; REF, referent; RV–, rotavirus negative; RV+, rotavirus positive; YR, year.

**Table 4.** Diarrhea Trends, International Centre for Diarrhoeal Disease Research, Bangladesh, Service Area Region (Model 2)

ISA	Feb 2000-March 2011 (prevaccine and CRT)	April 2011– March 2012 (YR1)			April 2012– March 2013 (YR2)			April 2013– March 2014 (YR3)			April 2014– September 2014 (YR3.5)			April 2011– September 2014 (vaccine years)		
		IRR	95%	CI	IRR	95%	CI	IRR	95%	CI	IRR	95%	CI	IRR	95%	CI
*0–12 months of age*																
RV+	REF	0.61	0.43	0.86	0.68	0.46	1.00	0.46	0.29	0.72	0.51	0.29	0.90	0.59	0.43	0.80
RV–	REF	1.07	0.74	1.52	1.33	1.05	1.69	1.14	0.87	1.49	0.89	0.62	1.28	1.15	0.91	1.47
*12–24 months of age*																
RV+	REF	0.72	0.44	1.17	0.58	0.29	1.12	0.74	0.41	1.33	0.33	0.11	0.99	0.65	0.42	1.02
RV–	REF	1.00	0.72	1.39	1.05	0.62	1.79	1.10	0.66	1.84	0.86	0.56	1.32	1.03	0.74	1.43
*0–24 months of age*																
RV+	REF	0.64	0.46	0.89	0.66	0.44	0.98	0.55	0.35	0.85	0.48	0.25	0.91	0.61	0.45	0.82
RV–	REF	1.05	0.80	1.38	1.27	0.98	1.64	1.12	0.85	1.46	0.89	0.70	1.13	1.12	0.91	1.37

Abbreviations: CI, confidence interval; CRT, cluster-randomized controlled trial; IRR, incidence rate ratio; ISA, International Centre for Diarrhoeal Disease Research, Bangladesh, service area; RV–, rotavirus negative; RV+, rotavirus positive; YR, year.

In Model 1, RV– diarrhea rates increased during the RCT period and the CRT period in both age groups. During periods of routine HRV use, there was an increased risk of RV– diarrhea in 0- to <12-month-old children (IRR 1.59, 95% CI 1.09–2.31) and no meaningful change in 12- to <24-month-old children. In Model 2, there were no statistically significant changes in RV– diarrhea rates during periods of HRV routine use.

### Diarrhea Incidence Trends: Government Service Area Villages

**Figure 4.**
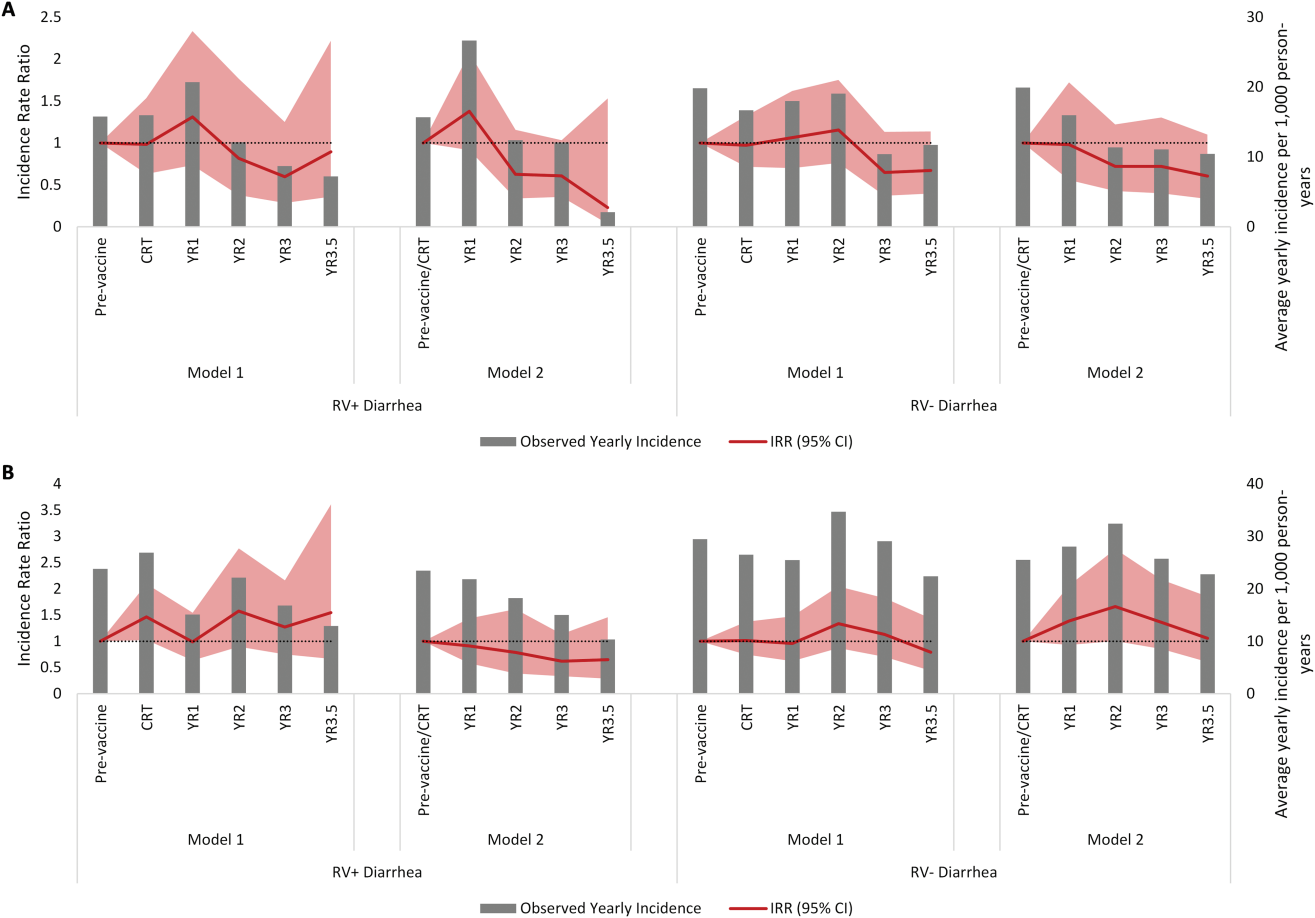
Observed incidence and IRRs of RV+ and RV– diarrhea of any severity presenting to Matlab Hospital in GSA villages using Models 1 and 2 in (*A*) 0 to <12-month-old children and (*B*) 12 to <24-month-old children. Abbreviations: CI, confidence interval; CRT, cluster-randomized controlled trial; GSA, government service area; IRR, incidence rate ratio; RV–, rotavirus negative; RV+, rotavirus positive; RCT, randomized, controlled trial; YR, year.

Using Model 1, with the pre-vaccine time period as the referent category, the incidence of RV+ diarrhea increased during the CRT period in 0- to <12-month-old children, but did not meaningfully change in 12- to <24-month-old children (Table 5; Figure 4). During periods of routine HRV use, there was an upward trend in the RV+ diarrhea incidence after each additional year of vaccine use in 0- to <12-month-old children, but no clear trends in 12- to <24-month-old children. During 3.5 years of routine use, there was no statistically significant change in the incidences of RV+ diarrhea in 0- to <12-month-old children (IRR 1.25, 95% CI 0.78–2.01) or in 12- to <24-month-old children (IRR 1.00, 95% CI 0.52–1.92). Using Model 2, there was a downward trend in the RV+ diarrhea incidence after each additional year of routine HRV use in both age groups (Table 6; Figure 4). However, during 3.5 years of routine HRV use, there was no meaningful change in the RV+ diarrhea rate in either age group. In Models 1 and 2, there were no statistically significant changes in RV– diarrhea rates during periods of HRV routine use.

**Table 5.** Diarrhea Trends, Government Service Area Region (Model 1)

GSA	Feb 2000– Oct 2008 (prevaccine)	Nov 2008– March 2011 (CRT)			April 2011– March 2012 (YR1)			April 2012– March 2013 (YR2)			April 2013– March 2014 (YR3)			April 2014– September 2014 (YR3.5)			April 2011– September 2014 (vaccine years)		
		IRR	95%	CI	IRR	95%	CI	IRR	95%	CI	IRR	95%	CI	IRR	95%	CI	IRR	95%	CI
*0–12 months of age*																			
RV+	REF	1.46	1.02	2.09	0.99	0.63	1.54	1.58	0.90	2.77	1.27	0.75	2.16	1.55	0.66	3.61	1.25	0.78	2.01
RV–	REF	1.01	0.75	1.37	0.96	0.62	1.47	1.33	0.87	2.04	1.13	0.70	1.82	0.79	0.43	1.43	1.10	0.73	1.65
RV+	REF	0.98	0.63	1.53	1.31	0.73	2.33	0.82	0.38	1.77	0.60	0.28	1.25	0.89	0.36	2.22	1.00	0.52	1.92
RV–	REF	0.97	0.72	1.32	1.06	0.70	1.62	1.16	0.76	1.75	0.65	0.37	1.13	0.67	0.40	1.14	0.98	0.64	1.50
*0–24 months of age*																			
RV+	REF	1.26	0.89	1.77	1.18	0.76	1.85	1.24	0.72	2.12	0.96	0.57	1.63	1.34	0.61	2.95	1.16	0.73	1.85
RV–	REF	0.98	0.77	1.26	0.99	0.71	1.38	1.25	0.88	1.78	0.92	0.61	1.40	0.75	0.47	1.19	1.04	0.74	1.47

Abbreviations: CI, confidence interval; CRT, cluster-randomized controlled trial; GSA, government service area; IRR, incidence rate ratio; RV–, rotavirus negative; RV+, rotavirus positive; YR, year.

**Table 6.** Diarrhea Trends, Government Service Area Region (Model 2)

GSA	Feb 2000–March 2011 (prevaccine and CRT)	April 2011–March 2012 (YR1)			April 2012–March 2013 (YR2)			April 2013–March 2014 (YR3)			April 2014–September 2014 (YR3.5)			April 2011–September 2014 (vaccine years)		
		IRR	95%	CI	IRR	95%	CI	IRR	95%	CI	IRR	95%	CI	IRR	95%	CI
*0–12 months of age*																
RV+	REF	0.91	0.58	1.43	0.79	0.39	1.61	0.62	0.34	1.14	0.65	0.29	1.46	0.78	0.48	1.27
RV–	REF	1.38	0.93	2.06	1.66	1.00	2.75	1.36	0.85	2.17	1.06	0.60	1.85	1.43	0.97	2.12
*12–24 months of age*																
RV+	REF	1.38	0.91	2.07	0.63	0.34	1.16	0.61	0.36	1.03	0.23	0.03	1.53	0.87	0.53	1.43
RV–	REF	0.98	0.56	1.72	0.72	0.43	1.22	0.72	0.40	1.30	0.61	0.33	1.10	0.79	0.51	1.23
*0–24 months of age*																
RV+	REF	1.12	0.80	1.55	0.72	0.48	1.08	0.62	0.41	0.94	0.50	0.24	1.06	0.82	0.57	1.19
RV–	REF	1.20	0.87	1.67	1.24	0.84	1.84	1.06	0.71	1.58	0.86	0.56	1.31	1.15	0.86	1.53

Abbreviations: CI, confidence interval; CRT, cluster-randomized controlled trial; GSA, government service area; IRR, incidence rate ratio; RV–, rotavirus negative; RV+, rotavirus positive; YR, year.

## DISCUSSION

Our study demonstrates a decreasing trend in RV+ diarrhea incidences among children <2 years of age from ISA villages presenting to Matlab Hospital during 3.5 years of routine HRV use. Using a conservative model to estimate pre-vaccination rotavirus diarrhea trends (Model 1), the results were not statistically significant. However, by restricting the analysis to control-only villages, we gained an additional 2 years of recent, pre-vaccine time to model baseline trends (Model 2), and found a statistically significant, 39% reduction in RV+ diarrhea rates in children 0 to <24 months of age. No significant impact of HRV on the RV+ diarrhea incidence among children from GSA villages was observed using either model. Differences in the population-level impacts between ISA and GSA villages are likely due to lower HRV coverage and lower reported diarrhea incidences in GSA areas, compared to ISA villages.

Our study also examined changes in the rate of RV– diarrhea as a control outcome, with the assumption that HRV introduction should have no significant impact on RV– diarrhea [22]. In Model 1, using only the pre-vaccine period in the referent category, we observed an increasing trend in both RV+ and RV– diarrhea rates in children 0 to <24 months of age in ISA villages during the RCT and CRT time periods. While other interventions or unmeasured biases may have influenced the all-cause gastroenteritis incidence, we believe this increase was due to changes in health-care–seeking behaviors due to the RCT. During the RCT, field staff visited the homes of infants enrolled in the study to remind parents to bring their child to the hospital for episodes of diarrhea [16]. A change in community health-care–seeking behavior is the most likely explanation, as there was no significant change in all-cause diarrhea in the corresponding time period in the GSA villages, where no RCT took place (Figure 1), and no specific pathogen was identified as a cause of the increase in all-cause diarrhea. The most conservative model to estimate the HRV impact (Model 1) modelled the RCT and CRT time periods separately and directly compared the pre-vaccine time period to the years of routine HRV use in both ISA and GSA villages. However, if increased health-care–seeking behaviors were sustained, results from Model 1 would underestimate the population-level impact of HRV.

In the secondary analysis (Model 2), both to increase power and to include relevant health-care–seeking behaviors to estimate the baseline incidence, we restricted the analysis to those villages randomized as control-only during the CRT period, and assessed the impact of routine HRV use on diarrhea over time. The referent category combined the pre-vaccine time period and the CRT time period. These models showed a significant impact of routine HRV use on RV+ diarrhea rates in 0- to <24-month-old children in ISA villages, but not in GSA villages. RV– diarrhea rates did not significantly change over time using this model. Notably, both models showed a decreasing trend in RV+ diarrhea in ISA villages during sustained HRV coverage. This analysis demonstrates the importance of using the appropriate baseline incidences and underlying trends in time-series analyses.

Despite the potential differences in health-care–seeking behavior over time, our results are similar to the RCT and CRT conducted in Matlab, Bangladesh, with the greatest impact of rotavirus vaccine on children 0 to <12 months of age. To our knowledge, no other population-level impact analyses have been reported in Asia with rotavirus diarrhea as the outcome, though a study in the Philippines saw a 60% (95% CI 55–64%) reduction in all-cause diarrhea hospitalizations within 4 years after rotavirus vaccine introduction [23]. Similar time-series analyses conducted 2–3 years after rotavirus introduction found a 49% (95% CI 32–63%) decrease in rotavirus diarrhea in <5-year-old children in Ghana [12], a 54% (95% CI 33–69%) decrease in rotavirus diarrhea in <1-year-old children in Malawi [11], a 33% (95% CI 25–41%) reduction in rotavirus diarrhea in <5-year-old children in Botswana [14], and a 38% reduction in rotavirus positivity among children <5 years old in Zambia [10]. Long-term impacts were also observed in Ghana [24] and Zambia [25]. Importantly, in these studies, >90% vaccine coverage for 1 or 2 doses of rotavirus vaccine were reported within 1 year of vaccine introduction. In our study, the maximum, 2-dose HRV coverage of 68% was attained in the ISA villages during the second year of routine use.

Our study has limitations. As in any time-series analysis, our study may have been confounded by other interventions or other unmeasured factors associated with RV+ diarrhea and the timing of the vaccine introduction. However, our confidence in the impact of HRV is increased, because no meaningful changes in RV– diarrhea incidences were observed. Second, while the Matlab HDSS database shows lower vaccine coverage in GSA areas, coverage may be underestimated or inaccurate due to the lack of recording on health cards in this region and potential reliance on maternal reports. Though measured with the same potential biases, during the study period, the average coverage for 3 doses of Diphtheria-Pertussis-Tetanus (DTP3) was 97% in ISA villages and 91% in GSA villages [26]. Third, with the available data, we were unable to assess the impact of the rotavirus vaccine on severe rotavirus diarrhea, as indicated by a Vesikari score ≥11, which is the outcome used in rotavirus vaccine clinical trials.

This study provides initial evidence of the population-level impact of rotavirus vaccines in children <2 years of age in regions of high vaccine coverage in Matlab, Bangladesh. Pecenka et al [27] estimated that, with a Gavi subsidy in Bangladesh, the averted cost/disability adjusted life year (DALY) ratio ranged between $58/DALY and $142/DALY, indicating a highly cost-effective vaccine, given 94% coverage of DTP3 in Bangladesh [27, 28] In our study, during the pre-vaccine period, rotavirus was detected in 34.5% of diarrhea cases in children <5 years of age presenting to Matlab Hospital. Other regions of Bangladesh show an average of 64% of diarrhea instances being due to rotavirus in children <5 years of age [29]. With sustained vaccine coverage and a considerable nationwide burden of rotavirus diarrhea, larger impacts of HRV on rotavirus gastroenteritis are likely to be observed long-term in Bangladesh. This may provide additional evidence to influence other countries in the region to introduce the rotavirus vaccine.

## Supplementary Data

Supplementary materials are available at *Clinical Infectious Diseases* online. Consisting of data provided by the authors to benefit the reader, the posted materials are not copyedited and are the sole responsibility of the authors, so questions or comments should be addressed to the corresponding author.

ciz133_suppl_Supplementary_Figure_1AB

ciz133_suppl_Supplementary_Figure_2AB

## Notes

***Acknowledgments.***The authors thank PATH for its commitment to their research efforts and for the negotiation of vaccines for Matlab, Bangladesh, following the cluster-randomized study. They thank the governments of Bangladesh, Canada, Sweden, and the United Kingdom for providing core/unrestricted support. They thank the families of the Matlab field area who participated in this study, and the field staff of the International Centre for Diarrhoeal Disease Research, Bangladesh, without whom they could not have completed this research. They thank PATH and GlaxoSmithKline for the donation of vaccines to the study and the study population, both during the cluster-randomized trial and for 3 years following the study.

***Financial support.***This work was supported by the Bill & Melinda Gates Foundation (grant number OPP1097672) and the National Institute of Allergy and Infectious Diseases (NIAID) of the National Institutes of Health (NIH) (grant number MERIT R37 AI032042).

***Potential conflicts of Interest.***All authors: No reported conflicts. All authors have submitted the ICMJE Form for Disclosure of Potential Conflicts of Interest. Conflicts that the editors consider relevant to the content of the manuscript have been disclosed.
