# Supplementary figures and images for: Impact of Rotavirus Vaccine Introduction in Children Less Than 2 Years of Age Presenting for Medical Care With Diarrhea in Rural Matlab, Bangladesh
Source: Clin Infect Dis. 2019 Feb 12;69(12):2059–70. doi: 10.1093/cid/ciz133 (PMC6880338; doi:10.1093/cid/ciz133)

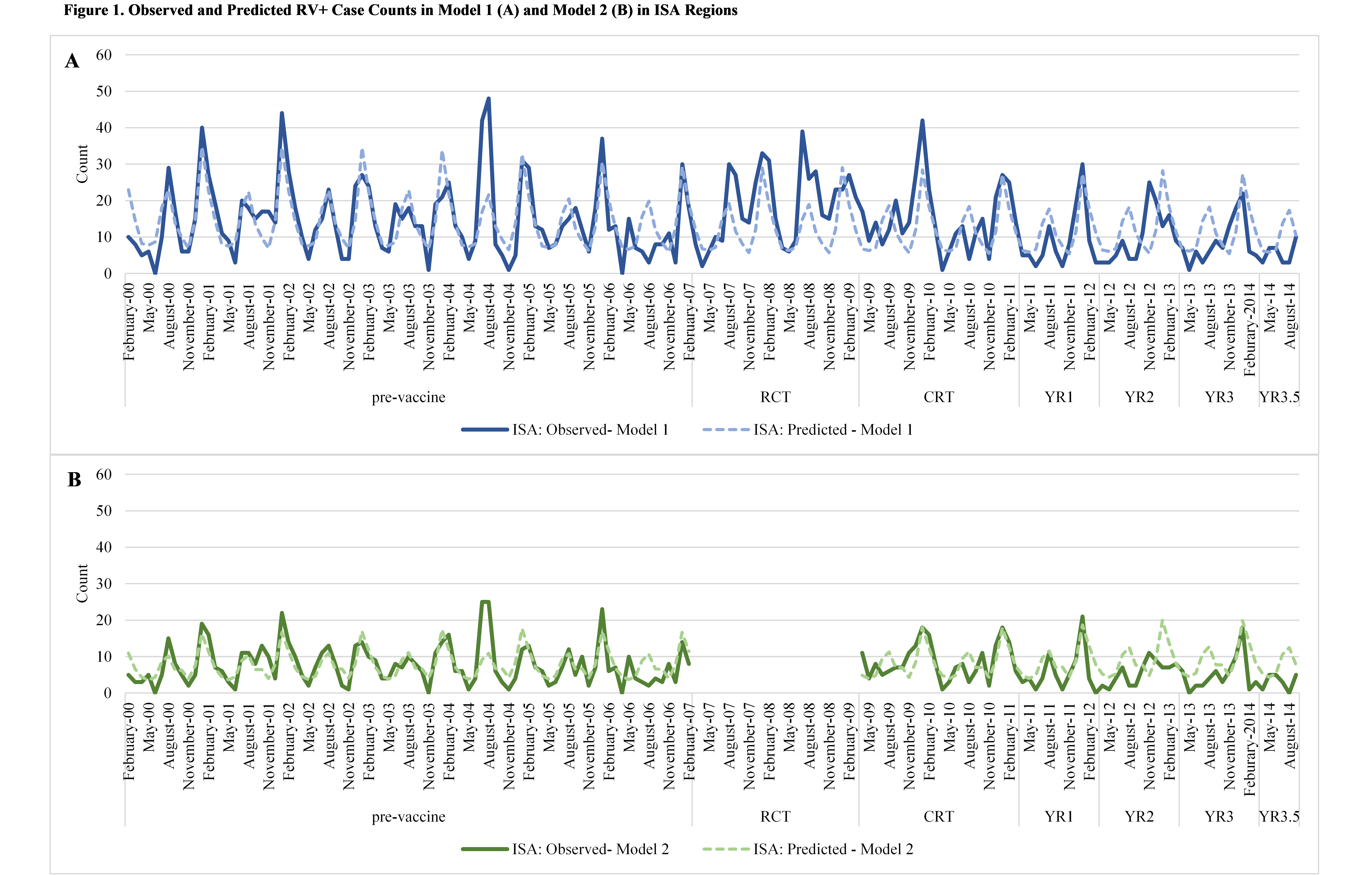

Supplement: ciz133_suppl_Supplementary_Figure_1AB [file ciz133_suppl_supplementary_figure_1ab.png]

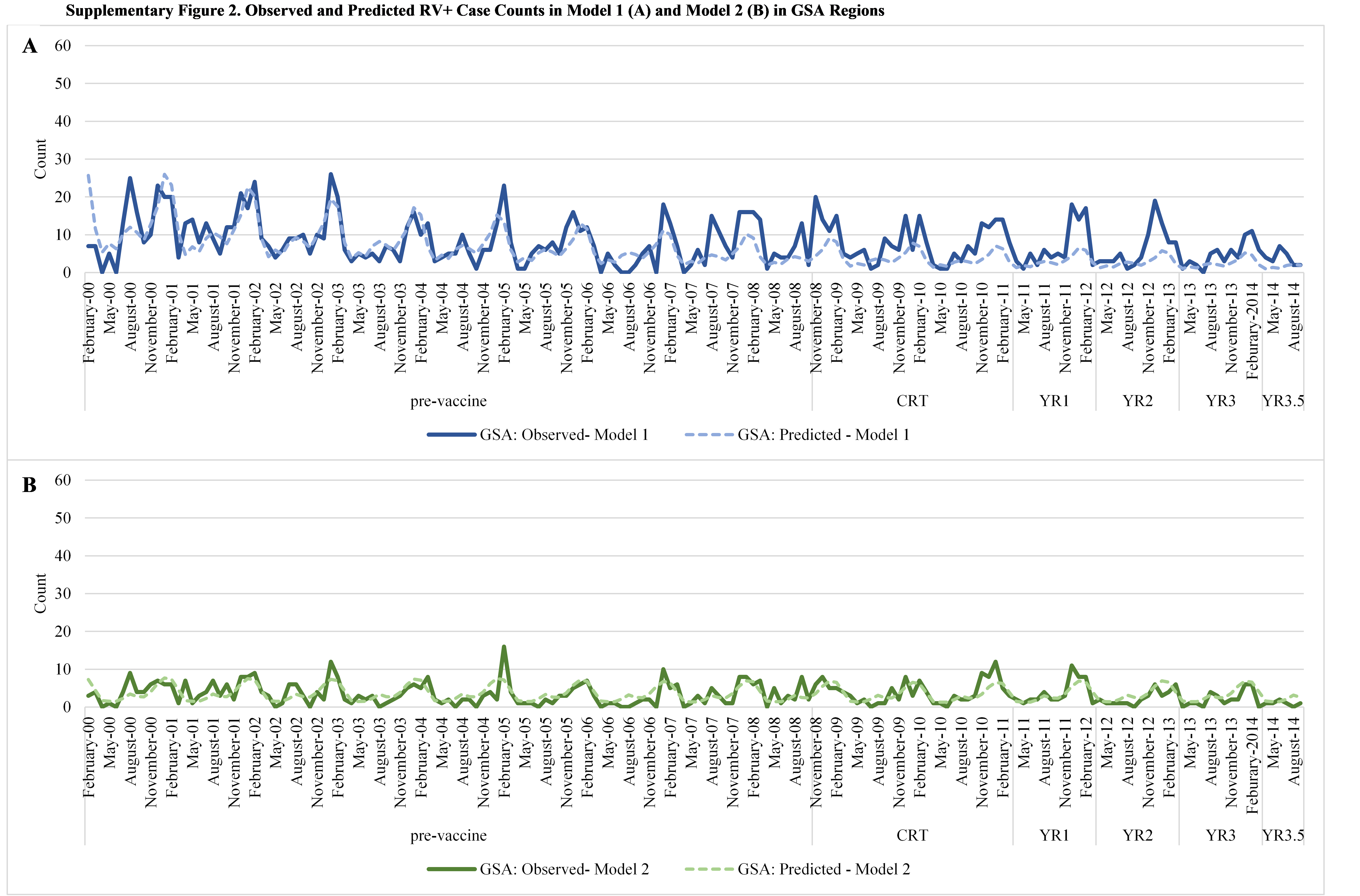

Supplement: ciz133_suppl_Supplementary_Figure_2AB [file ciz133_suppl_supplementary_figure_2ab.png]
